# DNA and recombinant adenovirus serotype 35 and 5 preventive HIV-1 vaccines with Env A inserts elicit cross-clade binding and V1V2 antibodies

**DOI:** 10.1186/1742-4690-9-S2-P136

**Published:** 2012-09-13

**Authors:** JD Fuchs, C Morgan, P Bart, N Kochar, N Frahm, E Swann, P Gilbert, S DeRosa, B Graham, G Nabel, H Liao, B Haynes, G Tomaras

**Affiliations:** 1 San Francisco Dept. of Public Health, San Francisco, CA, USA; 2 Fred Hutchinson Cancer Center, USA; 3 Centre Hospitalier Universitaire Vaudois, Switzerland; 4 Division of AIDS, NIH, USA; 5 Vaccine Research Center, NIH, USA; 6 Duke University, USA

## Background

We previously reported that combinations of DNA, recombinant adenovirus serotype 5 (rAd5) and 35 (rAd35) HIV-1 vaccines with clade A Env inserts elicit strong T-cell responses. Here we investigate their ability to induce cross-clade IgG binding antibody (bAb) responses. Based on recent evidence that Ab to the V1V2 loop in Env were correlated with reduced risk of HIV infection in the RV144 efficacy trial, we also evaluated those responses.

## Methods

HVTN 077 was a placebo-controlled, double-blinded trial that randomized 192 healthy, HIV-uninfected, Ad35 neutralizing antibody (nAb) seronegative participants into placebo (n=28) and 4 vaccine groups: rAd35/rAd5 (T1, n=34), DNA/rAd5 (T2, n=48), and DNA/rAd35 (T3, n=48) -- in persons seronegative for Ad5 nAb; and DNA/rAd35 (T4, n=34) seropositive for Ad5 nAb. Vaccines were given at 0,6 months (T1) and 0,1,2, and 6 months (T2-4). IgG bAb responses by multiplex assay against Group M Consensus (Con S), clade A (00MSA 4076), clade B (B.con.env03), and clade C (C.con.env03) gp140 and novel gp70 V1V2 scaffolds, V1V2 VRC A and V1V2 (Case A2), were measured 4 weeks post boost.

## Results

High frequency responses were elicited against clade A (T1 95.8%, T2 100%, T3 97.4%, T4 100%), clade B (T1 95.8%, T2 95.0%, T3 92.1%, T4 96.3%), clade C (T1 92%, T2 76%, T3 76%, T4 78%), and Con S (T1-4 100%). There were no significant between-group differences in response frequency or magnitude. The majority also had responses to V1V2 clade A (T1 100%, T2 87.8%, T3 83.8%, T4 85.2%) and clade B (T1 58.3%, T2 65.9%, T3 54.1%, T4 51.9%). The mean fluorescent intensity was higher in T2 vs T1 (p=0.005) for clade A V1V2. No significant differences were observed between other groups.

## Conclusion

All vaccine regimens tested elicited cross-clade bAb responses, including those that target V1V2. rAd-based HIV-1 vaccines, particularly using rare serotypes, warrant further development.

